# Impaired ion homeostasis as a possible associate factor in mucopolysaccharidosis pathogenesis: transcriptomic, cellular and animal studies

**DOI:** 10.1007/s11011-021-00892-4

**Published:** 2021-12-20

**Authors:** Lidia Gaffke, Zuzanna Szczudło, Magdalena Podlacha, Zuzanna Cyske, Estera Rintz, Jagoda Mantej, Karolina Krzelowska, Grzegorz Węgrzyn, Karolina Pierzynowska

**Affiliations:** Department of Molecular Biology, University of Gdansk, Wita Stwosza 59, 80-308 Gdansk, Poland

**Keywords:** Mucopolysaccharidosis, Ions homeostasis, Iron, Calcium, Zinc, Transcriptomics

## Abstract

Mucopolysaccharidoses (MPS) are a group of diseases caused by mutations resulting in deficiencies of lysosomal enzymes which lead to the accumulation of partially undegraded glycosaminoglycans (GAG). This phenomenon causes severe and chronic disturbances in the functioning of the organism, and leads to premature death. The metabolic defects affect also functions of the brain in most MPS types (except types IV, VI, and IX). The variety of symptoms, as well as the ineffectiveness of GAG-lowering therapies, question the early theory that GAG storage is the only cause of these diseases. As disorders of ion homeostasis increasingly turn out to be co-causes of the pathogenesis of various human diseases, the aim of this work was to determine the perturbations related to the maintenance of the ion balance at both the transcriptome and cellular levels in MPS. Transcriptomic studies, performed with fibroblasts derived from patients with all types/subtypes of MPS, showed extensive changes in the expression of genes involved in processes related to ion binding, transport and homeostasis. Detailed analysis of these data indicated specific changes in the expression of genes coding for proteins participating in the metabolism of Ca^2+^, Fe^2+^ and Zn^2+^. The results of tests carried out with the mouse MPS I model (*Idua*^−/−^) showed reductions in concentrations of these 3 ions in the liver and spleen. The results of these studies indicate for the first time ionic concentration disorders as possible factors influencing the course of MPS and show them as hypothetical, additional therapeutic targets for this rare disease.

**Supplementary Information:**

The online version contains supplementary material available at 10.1007/s11011-021-00892-4.

## Introduction

Mucopolysaccharidoses (MPS) are caused by genetic defects in lysosomal enzymes, leading to the accumulation of partially undegraded glycosaminoglycans (GAG) in tissues and organs (Tomatsu et al. 2018). GAG are complex unbranched polysaccharides that play important roles in binding of growth factors to their receptors on the cell surface, and also determine the flexibility of connective tissue. In a healthy organism, GAG chains are cut into smaller fragments in specific places by several lysosomal enzymes from the group of endoglucuronidases or endohexosaminidases, and their actions are strictly correlated. Deficiency of individual enzymes, resulting in accumulation of given GAG types, is the basis for the division of MPS into 11 types (Table 1). All MPS types/subtypes are inherited in autosomal recessive manner with the exception of MPS II, which is an X-linked disease. MPS occurs at a frequency of not more than 1: 25,000 live births (Tomatsu et al. 2018).

**Table 1:** Characteristics of MPS types/subtypes

MPS subtype	Common name	Stored GAG	Affected gene	Deficient enzyme
MPS I	Hurler, Scheie, Hurler-Scheie syndrome	HS, DS	*IDUA*	α-L-Iduronidase
MPS II	Hunter syndrome	HS, DS	*IDS*	Iduronate sulfatase
MPS IIIA	Sanfilippo syndrome	HS	*SGSH*	Heparan sulfamidase
MPS IIIB	Sanfilippo syndrome	HS	*NAGLU*	N-Acetylglucosaminidase
MPS IIIC	Sanfilippo syndrome	HS	*HGSNAT*	Acetyl-CoA:α-glucosaminide N-acetyltransferase
MPS IIID	Sanfilippo syndrome	HS	*GNS*	N-Acetylglucosamine 6-sulfatase
MPS IVA	Morquio syndrome	KS, CS	*GALNS*	Galactose-6-sulfate sulfatase
MPS IVB	Morquio syndrome	KS	*GLB1*	β-Galactosidase
MPS VI	Maroteaux–Lamy syndrome	DS.	*ARSB*	N-Acetylgalactosamine-4-sulfatase
MPS VII	Sly syndrome	HS, DS, CS	*GUSB*	β-Glucuronidase
MPS IX	Natowicz syndrome	H	*HYAL1*	Hyaluronidase

*HS* heparan sulfate, *DS* dermatan sulfate, *CS* chondroitic sulfate, *H* hyaluronic acid

Depending on the type of MPS, the symptoms of the disease may vary. Variability in the disease course is related to the differences in the activity of deficient enzyme (the presence of residual activity or complete inactivity), kind(s) of stored GAG, the efficiency of GAG synthesis (which is a strictly individual feature), and number of other as yet incompletely known factors, such as redox economy, individual tendency to express inflammation, and others (Jakóbkiewicz-Banecka et al. 2014). General symptoms affecting all types of MPS include hepatomegaly, coarse facial features, dysostosis multiplex leading to short stature with disproportionately short neck and trunk, dysplasia, thickened skin (except MPS IV), and hernias. Patients are also characterized by frequent progressive joint stiffness (MPS I, II, VI) and carpal tunnel syndrome as well as recurrent respiratory and ear infections, adenotonsillar hypertrophy/upper airway obstruction. Valvular heart disease, cardiac hypertrophy and/or valves disease, and central nervous system involvement or neurological abnormalities occur depending on the type of disease, though metabolic brain disorders are characteristic for most of them (MPS types IVA, “classical IVB”, VI, and IX are exceptions) (Tomatsu et al. 2018).

There are various therapeutic strategies for MPS, including enzyme replacement therapy (ERT), hematopoietic stem cell transplantation or experimental substrate reduction therapy and gene therapy. Undoubtedly, ERT which consists in delivering the active form of the missing enzyme to the patient’s body, available for MPS I, II, IVA, VI and VII, is the most often used therapy, as it is effective in reducing urinary GAG levels and liver and spleen volume (Noh and Lee 2014; Fecarotta et al. 2018; Gaffke et al. 2018).

However, in patients undergoing ERT, a number of disease symptoms are noted which do not improve despite the normalization of GAG levels. One obvious problem is limited penetration into specific tissues of the organism, such as the central nervous system and sensory organs or the skeletal system. Nevertheless, other symptoms are also hard to eliminate by ERT. For example, in patients with MPS, despite enzyme administration and lowering the GAG levels, no improvement was noted in the respiratory (spirometric parameters), cardiological (mitral valve regurgitation, aortic valve regurgitation, muscle hypertrophy), and otolaryngological functions, as well as shoulder flexion, symptoms of nocturnal hypoventilation and sleep apnea, visual acuity impairment due to corneal clouding, and bone mineral density (Muenzer 2014; Bik-Multanowski et al. 2017; Pérez-López et al. 2017; Sawamoto et al. 2020). A detailed description of disorders improving and not improving in patients with different types of MPS under the influence of ERT was compiled by Parini and Deodato (2020). These clinical observations suggest the occurrence of additional factors contributing to the pathogenesis of the disease, modulating its course, which are not dependent on GAG storage, or which, once formed, will not be reversed despite the removal of GAG storage.

Metal ions play important roles in the human body as secondary messengers or active sites of metalloenzymes, and take part in the stabilization of the configuration of proteins and nucleic acids. The phenomenon of disturbing the levels of metals (mainly iron, zinc and copper) by influencing changes in proteostasis also accompanies the death of nerve cells. Their participation in synaptic transmission has also been proven, where Zn^2+^ ions, released together with glutamate from glutamatergic vesicles, may play an important role in preserving memory. It is worth mentioning that the ability of some of the metal ions to exist in different valence states is necessary to capture, store, transport and utilize oxygen to maintain an equilibrium state that prevents oxidative stress in cells, which is now considered to be the main cause of aging (Barnham and Bush 2014). Moreover, it turns out that disturbances in the concentration of metal ions can lead to changes in the regulation of gene expression (Rashid et al. 2003, Rosati and McKinnon (2004). Therefore, ion imbalance can be the main or additional cause of many human diseases.

Studies on ionic disorders in MPS, reported to date, were scarce and concerned only calcium ions in individual MPS types (Pereira et al. 2010; Salvalaio et al. 2017). Our studies, for the first time, engage comprehensive analysis of ion imbalance (Ca^2+^, Zn^2+^, Fe^2+^), from transcriptomic, through cellular, to mouse model studies. We propose that uncovered ion homeostasis disturbances are one of the factors, beside GAG accumulation, influencing pathogenesis of this disease.

## Materials and methods

### Cell lines and cell cultures

Fibroblast lines collected from patients with all MPS types (Table 2) and HDFa (Human Dermal Fibroblasts, adult) line were purchased from the Coriell Institute. Cells were grown under standard conditions, at 37 °C, 95% humidity with an atmosphere saturated with 5% CO_2_ in DMEM medium supplemented with 10% Fetal Bovine Serum (FBS), and in the presence of antibiotics.

**Table 2:** Characteristics of MPS patient-derived fibroblasts

MPS subtype	Sex^(a)^	Age^(b)^	Mutated gene	Mutations	Cat. No.
I	F	1	*IDUA*	p.Trp402X/p.Trp402X	GM00798
II	M	3	*IDS*	p.His70ProfsX29/−	GM13203
IIIA	F	3	*SGSH*	p.Glu447Lys/p.Arg245His	GM00879
IIIB	M	7	*NAGLU*	p.Arg626Ter/p.Arg626Ter	GM00156
IIIC	M	8	*HGSNAT*	p.Gly262Arg/pArg509Asp	GM05157
IIID	M	7	*GNS*	p.Arg355Ter/p.Arg355Ter	GM05093
IVA	F	7	*GALNS*	p.Arg386Cys/p.Phe285Ter	GM00593
IVB	F	4	*GLB1*	p.Trp273Leu/p.Trp509Cys	GM03251
VI	F	3	*ARSB*	ND	GM03722
VII	M	3	*GUSB*	p.Trp627Cys/p.Arg356X	GM00121
IX	F	14	*HYAL1*	ND	GM17494

(a) *F* female, *M* male; (b) age at the time of sample collection; ND, not determined

### Animals

A mouse model of MPS I (*Idua*^−/−^) was purchased from the Jackson Laboratories. The animals were kept as an inbreeding colony of heterozygous individuals with constant access to water and food under standard conditions (temperature 22 °C, humidity 50–55%, day/night cycle 12 h/12 h). *Idua*^−/−^ (*n* = 8) and *Idua*^+/+^ (*n* = 8) males were euthanized at age of 6 months with Morbital (2 ml/kg), and organs were harvested. All experiments were conducted in accordance with the guidelines of the Council of the European Communities (2010/63/EU) and after approval by the Local Ethics Committee for Animal Experiments in Bydgoszcz (Poland).

### Transcriptomic studies

#### RNA isolation and purification

5 × 10^5^ cells were passaged in 10 cm diameter-plates and allowed to attach overnight. Cells were lysed with a solution containing guanidine isothiocyanate and β-mercaptoethanol (to effectively inactivate RNAse) and homogenized using a QIAshredder column. Next, RNA was extracted with the RNeasy Mini kit (Qiagen, Hilden, Germany) and treated with Turbo DNase (Life Technologies, Life Technologies, Carlsbad, CA, USA) in accordance with the manufacturer’s instructions. The quality of the isolated RNA samples was assessed by Nano Chips RNA (Agilent Technologies, Santa Clara, CA, USA) in the Agilent 2100 Bioanalyzer System. Genetic material from each cell line was isolated in 4 independent replications.

#### RNA-seq analysis

The mRNA libraries were generated with Illumina TruSeq Stranded mRNA Library Prep Kit and the cDNA libraries were sequenced on a HiSeq4000 (Illumina, San Diego, CA, USA) (PE150 (150 bp paired-end); 40 million of raw reads; 12 Gb of raw data per sample). FastQC (version v0.11.7) was used to quality assessment of data. Raw readings were mapped to the GRCh38 human reference genome from the Ensembl database with the use of Hisat2 v. 2.1.0 program. The Cuffquant and Cuffmerge programs (version 2.2.1) and the GTF Homo_sapiens.GRCh38.94.gtf file from the Ensembl database (https://www.ensembl.org/index.html as of August 20, 2021) were used to calculate the expression level of the transcripts. The Cuffmerge program was started with the library-norm-method classic-fpkm parameter, normalizing the expression values by means of the FPKM algorithm. The RNA-seq data were submitted to the NCBI Sequence Read Archive (SRA), and the accession no. is PRJNA562649.

#### Statistical analysis

R software v3.4.3 was used to performed statistical analyses. One-way analysis of variance (ANOVA) on log_2_(1 + x) values which have normal continuous distribution, and post hoc Student’s t test with Bonferroni correction or Benjamini–Hochberg method were used to analyze statistical significance between two groups or the false discovery rate (FDR), respectively. Transcript classification was performed with the use of the Ensembl gene database (the BioMart interface; https://www.ensembl.org/info/data/biomart/index.html, as for August 20, 2021). The procedure of RNA isolation and transcriptomic analyzes using the RNA-seq technique has already been described in detail in our previous work (Gaffke et al. 2020).

### Determination of the selected metal ions’ concentrations

Following kits were used for determination of concentrations of calcium, iron, and zinc ions: Calcium Assay Kit (Colorimetric) (Abcam, ab102505), Iron Assay Kit (Colorimetric) (Abcam, ab83366), and Zinc Assay Kit (Abcam, ab102507), respectively. In cellular analyses, 6 × 10^5^ cells were passaged in 10 cm diameter-plates and allowed to attach overnight. Cells were then harvested and transferred to tubes, washed with the PBS buffer (137 mM NaCl; 2.7 mM KCl; 10 mM Na_2_HPO_4_; 1.8 mM KH_2_PO_4_), and centrifuged (300 x *g* for 10 min). The experiment was performed in 3 independent replications. In animals tissues analysis, 10 mg of brain, liver or spleen were separated and washed with the PBS buffer. Cellular pellets and tissues were next lysed/homogenized for 3 h with Calcium Assay Buffer, Iron Assay Buffer or EDTA-free lysis buffer (0.9% NaCl; 0.5% Triton X-100; 0.1% SDS; 1% sodium deoxycholate; 50 mM Tris-HCl pH 7.5) to determine concentrations of calcium, iron or zinc ions, respectively. To obtain cellular lysate or tissue homogenate, samples were centrifuged at 16,000 x *g* for 10 min. Calcium, iron or zinc ion concentrations were assessed by colorimetric measurements and appropriate calculations, according to the manufacturer’s instructions.

### Measurement of the GAG level

Levels of GAG in the obtained cell lysates and in samples of mouse urine was measured using the Glycosaminoglycan Assay Blyscan™ (Biocolor Life Science Assays) kit. Urine samples were collected from mice prior to initiating the euthanasia procedure or during organ harvesting directly from the bladder. The GAG level assessment was carried out according to the manufacturer’s instructions.

## Results

Transcriptomic studies, indicating disturbances in expression of genes whose products are involved in maintaining ion balance, were performed with fibroblasts collected from patients with all types/subtypes of MPS (MPS I, II, IIIA, IIIB, IIIC, IIID, IVA, IVB, VI, VII, IX) and wild-type fibroblasts (HDFa, Human Dermal Fibroblasts, adults) using the RNA-seq technique. In this analysis, transcripts involved in ion binding (GO:0043167), ion transport (GO:0006811) and ion homeostasis (GO:0006873) were selected (the selection was made on the basis of the Ensembl database as of April 1, 2021).

**Fig. 1:**
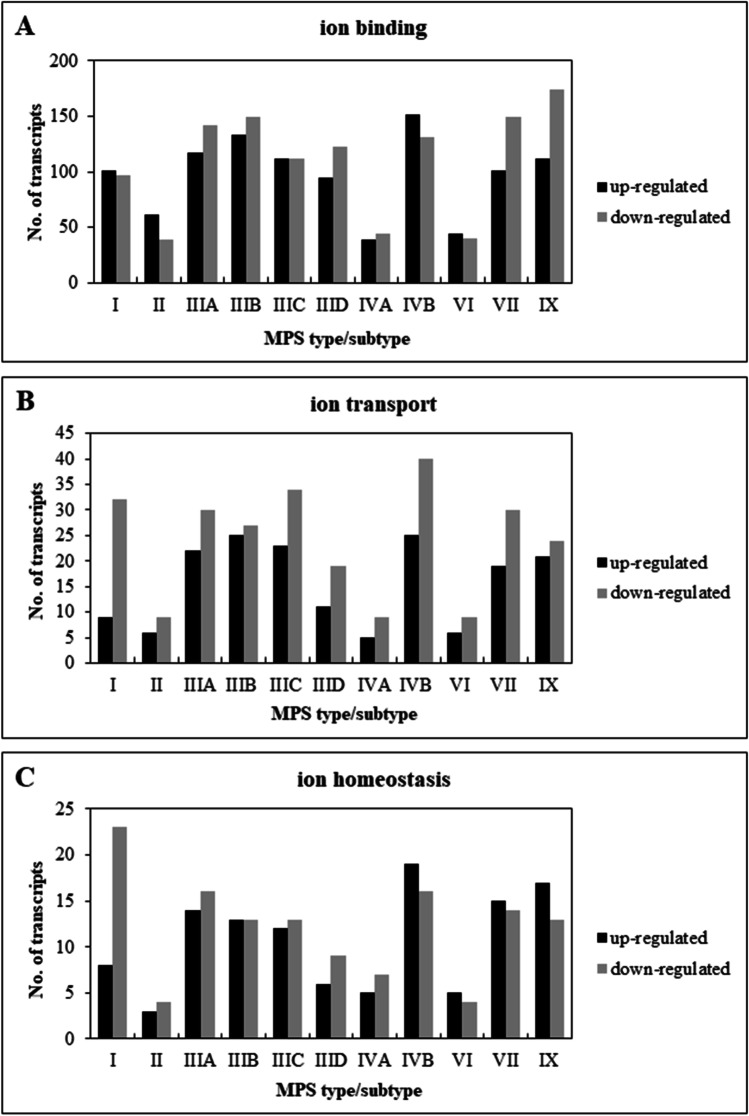
Number of up- and down- regulated transcripts included into ion balance-related GO terms which are ‘ion binding’ (GO:0043167), ‘ion transport’ (GO:0006811) and ‘ion homeostasis’ (GO:0006873) in different types/subtypes of MPS relative to control cells (HDFa)

The first stage of the research aimed at indicating the importance of the phenomenon of ion balance disorders showed an unexpectedly large number of transcripts which products are involved in the above-mentioned processes, and whose expression level was disturbed in MPS cells as compared to wild-type cells. This number for ion binding was over 200 for some MPS types/subtypes (MPS IIIA, IIIB, IIIC, IIID, IVB, VII, IX). For ion transport and ion homeostasis, the number was not so high, remaining at 45 and 20 altered expression transcripts, respectively (Fig. 1).

**Fig. 2:**
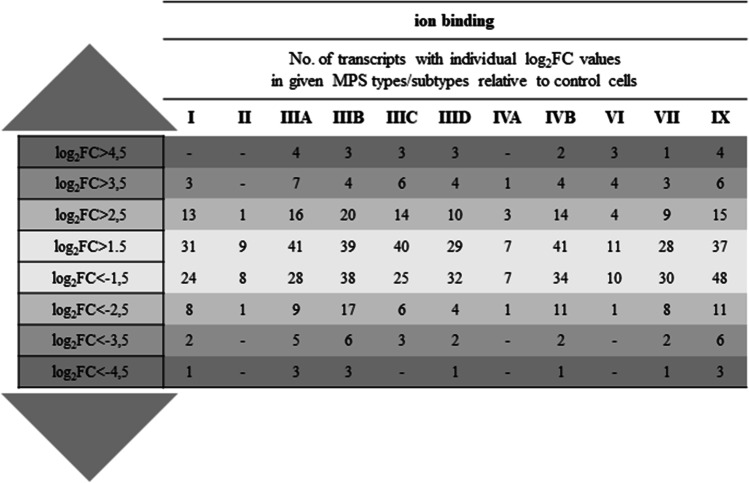
Number of transcripts included into ‘ion binding’ (GO:0043167) term with altered expression depending on the level of fold-change value (log_2_FC) in different types/subtypes of MPS relative to control cells (HDFa)

**Fig. 3:**
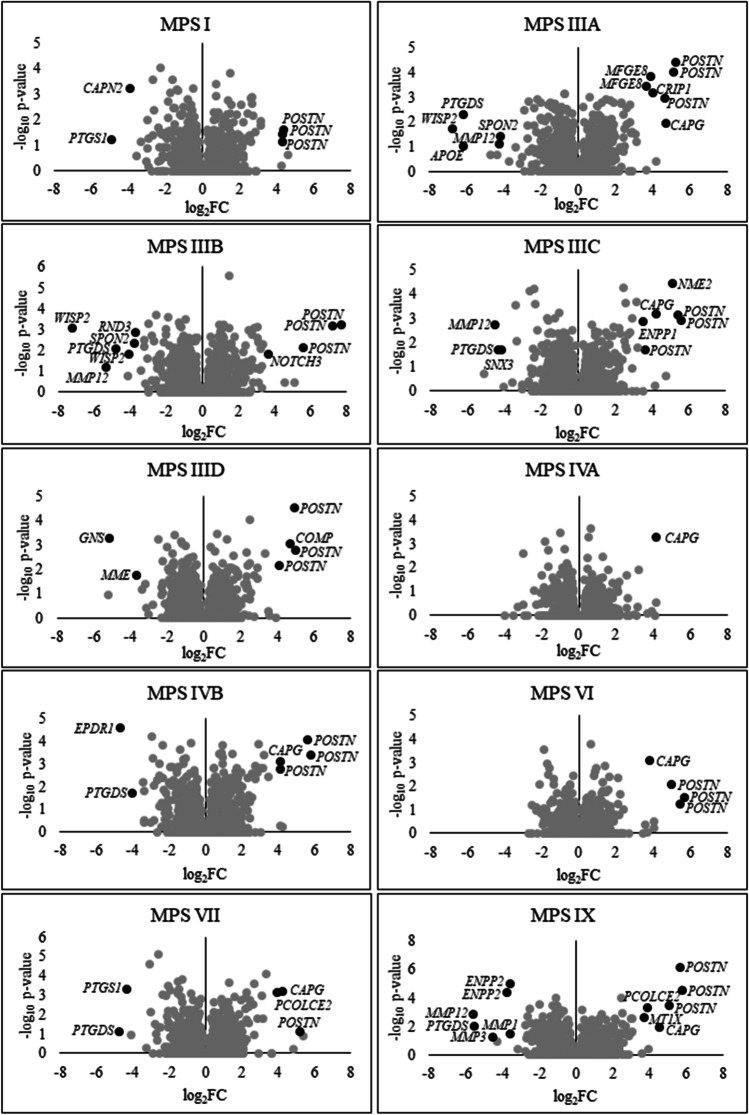
Volcano plots indicating transcripts which expression is significantly changed (log_2_FC > 3.5), included into ‘ion binding’ (GO:0043167) term in different types/subtypes of MPS relative to control cells (HDFa); no such transcripts have been reported in the case of MPS II

The number of ion balance-dependent transcripts with particularly high values of the fold change of expression level in MPS cells compared to wild-type cells was also analyzed. Within the ion binding process, in some types/subtypes of MPS, the number of transcripts with over 3-fold altered expression (log_2_FC > 1.5) exceeded 70, and transcripts with over 12-fold altered expression (log_2_FC > 3.5) exceeded 10 (Fig. 2). For ion transport and ion homeostasis, the number of transcripts with over 3-fold altered expression (log_2_FC > 1.5) oscillated around 10 (Fig. S1). Selection of ion balance-dependent transcripts with values of the fold change in expression level exceeding 12 (log_2_FC > 3.5) and high values of statistical significance between MPS and wild-type cells led to the identification of 34 such transcripts, among which prostaglandin synthase (*PTGDS*) and matrix metallopeptidase (*MMP12*) (down-regulated) as well as periostin (*POSTN*) and capping actin protein (*CAPG*) (up-regulated) were the most frequently repeated in different types/subtypes of MPS (Fig. 3 and Figs. S2-S4).

Transcripts were also selected for those which expression level is disturbed in many types/subtypes of MPS as compared to control cells simultaneously. The 21 transcripts with expression levels deviating from this level in healthy cells of at least 8 MPS types/subtypes are indicated in Table 3. Among them, there were transcripts which products are components of signaling pathways (*STK32B, MAP2K1, CRELD1, NOTCH3*), conditioning the proper functions of transport channels (*HEPH, TMEM38B*), regulating the functions of the cytoskeleton (*CAPG, CDH2*), transcription factors (*WTIP, FHL3*) and members of the extracellular matrix (*POSTN*).

**Table 3:** Ion-related transcripts with especially high fold change of expression level in MPS cell lines related to HDFa cell line

Transcript	log_2_FC of selected transcript expression in particular MPS type vs. HDFa line^(a)^										
	I	II	III A	III B	III C	III D	IVA	IVB	VI	VII	IX
ion binding											
*POSTN*	**4.38**	4.27	**5.27**	**7.26**	**5.42**	**5.01**	3.70	**5.69**	**5.47**	**5.20**	**5.83**
*POSTN*	**4.34**	4.42	**5.14**	**7.75**	**5.63**	**4.98**	4.14	**5.84**	**5.75**	5.38	**5.71**
*POSTN*	**4.33**	3.83	**4.68**	**5.63**	**3.67**	**4.16**	3.01	**4.16**	**5.04**	4.86	**5.13**
*CAPG*	**2.73**	2.98	**4.71**	**1.98**	**4.27**	**2.75**	**4.17**	**4.14**	**3.82**	**4.28**	**4.55**
*PCOLCE2*	**2.63**	4.37	**3.14**	**3.22**	**2.46**	**2.91**	**3.21**	**3.21**	3.46	**3.99**	**3.93**
*CDH2*	1.78	**2.11**	**1.04**	**3.05**	**2.56**	1.69	**2.05**	**2.94**	1.44	**1.10**	**2.98**
*NOTCH3*	**2.42**	0.97	**1.93**	**3.66**	**2.56**	**2.93**	2.67	**1.97**	2.01	**3.32**	**2.70**
*STK32B*	**2.19**	**1.66**	1.75	**1.19**	**1.58**	**2.31**	1.54	**1.96**	**1.62**	0.06	**2.62**
*HEPH*	**1.93**	**1.97**	1.37	1.29	**1.98**	**2.48**	**1.59**	**1.37**	1.81	**2.66**	**1.50**
*GALNT10*	**0.53**	0.74	**0.66**	**1.13**	**0.67**	**0.79**	0.54	**0.91**	0.27	**0.86**	**0.85**
*WTIP*	**0.65**	**0.64**	**0.79**	**0.61**	**0.84**	**0.68**	0.88	**0.96**	0.51	0.67	**0.73**
*FHL3*	**0.72**	**0.93**	**0.78**	**0.50**	0.84	0.50	0.92	**0.67**	**0.84**	**1.38**	**0.72**
*SAR1A*	*−0.61*	*−0.55*	*−0.68*	*−0.61*	*−0.69*	−0.31	*−0.74*	*−0.64*	−0.46	*−0.46*	*−0.48*
*USP12*	*−0.77*	*−0.62*	*−0.73*	−0.34	*−0.62*	*−0.73*	*−0.66*	*−0.63*	−0.61	*−0.71*	*−0.71*
*MAP2K1*	*−0.53*	*−0.63*	*−0.83*	−0.73	*−0.77*	*−0.67*	*−0.54*	*−0.59*	−0.52	−0.49	*−0.84*
*CRELD1*	−0.58	*−1.29*	*−1.28*	−0.52	*−1.04*	*−1.03*	*−1.40*	*−1.80*	*−1.58*	−0.03	*−1.40*
*PLCB4*	*−1.53*	*−1.82*	*−0.93*	*−3.07*	*−1.65*	−1.00	*−1.65*	*−2.96*	*−1.63*	*−0.86*	*−1.97*
*ENPP2*	*−1.34*	−0.97	*−2.35*	*−2.79*	*−2.39*	*−1.70*	−1.82	*−2.55*	−1.49	*−1.39*	*−3.52*
*SNX25*	*−0.68*	*−0.90*	*−0.83*	0.04	*−0.72*	−0.21	*−0.63*	*−0.49*	−0.65	*−0.70*	*−0.62*
*DYNC1LI1*	*−0.56*	*−0.55*	*−0.73*	*−0.77*	*−0.62*	−0.52	−0.42	*−0.63*	−0.36	*−0.58*	*−0.76*
ion transport/ion homeostasis											
*HEPH*	**1.93**	**1.97**	1.37	1.29	**1.98**	**2.48**	**1.59**	**1.37**	1.81	**2.66**	**1.50**
*TMEM38B*	*−0.90*	*−0.87*	*−1.34*	*−1.09*	*−1.12*	*−1.44*	−1.09	*−0.86*	−0.47	*−1.18*	*−0.99*

(a) Bold and italic fonts indicate up-regulated and down-regulated transcripts, respectively, while statistically insignificant differences are presented in a regular font

**Fig. 4:**
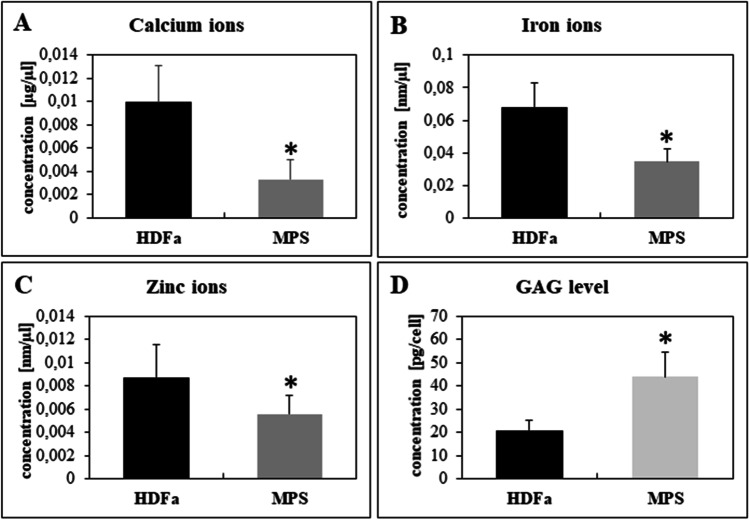
Concentration of calcium (**A**), iron (**B**), and zinc (**C**) ions as well as the level of GAG (D) in MPS I patient-derived fibroblasts relative to control cells (HDFa). Data are shown as the mean of 3 independent replicates. Error bars represent standard deviation. Asterisks indicate statistically significant differences between the groups at the significance level *p* < 0.05

Since the activities, levels or functions of proteins encoded by these transcripts are related to the levels of metal ions, mostly Ca^2+^ (*MMP12, CAPG, CDH2, STK32B, SAR1A, CRELD1, PLCB4, ENPP2, TMEM38B*), Zn^2+^ (*MMP12, WTIP, FHL3, USP12, ENPP2*) and Fe^2+^ (*HEPH*), we decided to test the concentrations of these three metal ions in cell cultures, using fibroblasts derived from a patient with MPS type I, revealing a severe phenotype (Hurler syndrome). The results of these experiments showed decreased concentrations of Ca^2+^, Zn^2+^ and Fe^2+^ relative to control cells; as expected, an increase in GAG level was evident in MPS I cells (Fig. 4).

**Fig. 5:**
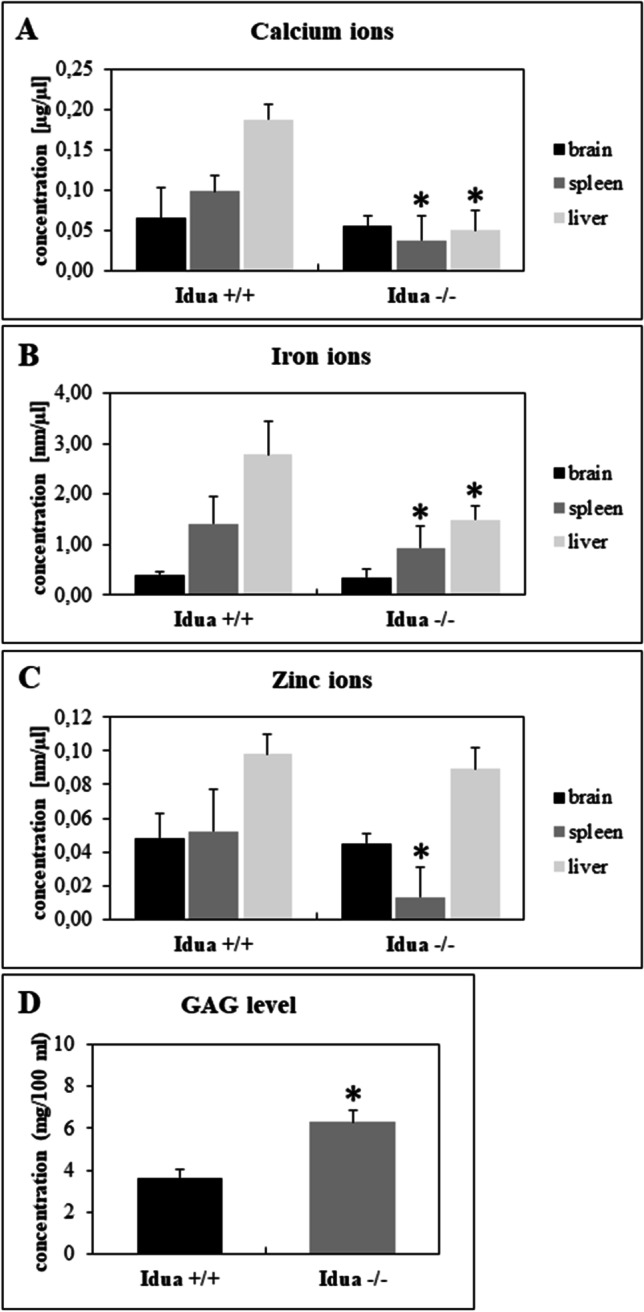
Concentration of calcium (**A**), iron (**B**), and zinc (**C**) ions as well as the level of GAG (D) in different organs in *Idua*^−/−^ mice (*n* = 8) relative to *Idua*^+/+^ mice (*n* = 8). Error bars represent standard deviation. Asterisks indicate statistically significant differences between the groups at the significance level *p* < 0.05

To test levels on investigated ions in animals, the experiments were conducted with the MPS I mouse model. Reduced concentrations of Ca^2+^ and Fe^2+^ were observed in the liver and spleen of MPS I mice, but not in the nervous tissue, relative to wild-type mice. On the other hand, a decreased level of Zn^2+^ was noted only in the spleen of MPS I mice (Fig. 5).

## Discussion

The overall efficacy of enzyme replacement therapy (ERT) for MPS is difficult to assess, mainly due to the lack of sufficient data in clinical trials that have so far led to inconclusive results (Kuiper et al. 2019). However, a growing body of evidence indicates that ERT, considered the simplest and the most appropriate of the proposed therapies for this disease, is not as effective as expected (see Introduction for details). It is worth noting that changes which are not improved with ERT affect many tissues and organs of the organism, not just those that are not reached by the enzyme, as has been commonly supposed. As mentioned previously, this phenomenon may suggest that the presented symptoms are not solely dependent on GAG storage, but also arise from as yet unidentified aspects of MPS pathogenesis. Alternatively, these changes once formed will not be reversed despite normalization of GAG levels.

One of the processes that play a key role in maintaining the organism’s homeostasis is the ion balance. Disturbances in the levels of metal ions are observed in many diseases, most of which is related to the dysfunction of ion channels, i.e. channelopaties. These include diseases of almost all organism systems, like the nervous system (e.g. generalized epilepsy with febrile seizures plus and episodic ataxia), the cardiovascular system (e.g, QT syndrome), the respiratory system (e.g, cystic fibrosis), the endocrine system (e.g, familial hyperinsulinemic hypoglycemia), the urinary system (e.g, Bartter syndrome), and the immune system (e.g, myasthenia gravis, neuromyelitis optica) (Kim 2014). Significant roles of metal ions have also been observed in the course of neurodegenerative diseases such as Alzheimer’s, Parkinson’s, Huntington’s diseases or amyotrophic lateral sclerosis (Gaeta and Hider 2005; Barnham and Bush 2014), where they influenced the efficiency of aggregation of proteins such as β-amyloid and hyperphosphorylated tau protein or modulation of the level of reactive oxygen species. Interestingly, recent reports demonstrated changes in the expression of genes encoding ion channels in breast cancer (Ko et al. 2013), follicular lymphoma (Magi et al. 2019) and different solid tumors (Biasiotta et al. 2016).

Since increasing number of studies indicate significant roles of metal ions in the pathogenesis of many diseases (see preceding paragraph), and because there is little data about this aspect of MPS, the aim of this work was to determine changes related to the ion balance in fibroblasts collected from patients, as well as in a mouse model of type I of this disease. We analyzed both the expression profiles of ion balance-related transcripts and the concentrations of these ions in cultured cells and in animal organs.

The changes in the expression of ion balance-related transcripts noted in this study were surprisingly large, covering about 300 such transcripts (Fig. 1), including transcripts with disturbances in the expression level in many types/subtypes of MPS (Table 3) or disorders with particularly high values of fold changes between MPS and control groups (Figs. 2 and 3, and Figs. S1-S4). Such results do not leave doubts about the significant role of these ion homeostasis disorders in the pathogenesis of MPS. Dysregulation of calcium signaling pathway was also indicated by transcriptomic analyzes carried out in nervous tissue on the MPS II mouse model (Salvalaio et al. 2017).

As most of the detected impairments in genes’ expressions were associated with Ca^2+^, Fe^2+^ and Zn^2+^, we decided to test concentrations of these three metal ions in cells taken from patients and in various organs of MPS I mice. Reduced levels of tested ions in MPS I fibroblasts, and decreased concentrations of Ca^2+^, Fe^2+^ and Zn^2+^ in spleen, and those of Ca^2+^, Fe^2+^ in liver were detected in MPS I mice (Figs. 4 and 5). Levels of Ca^2+^ ions in MPS I cells were also investigated by Pereira et al. (2010). They demonstrated that in isolated splenic lymphocytes of MPS I mice, the release of calcium ions from endoplasmic reticulum (ER) and lysosomes was enhanced relative to wild-type control, after treatment with thapsigargin (a specific inhibitor of Ca^2+^-ATPase from the ER) or bafilomycin A (an inhibitor of lysosomal H+/ATPase). Therefore, they concluded that calcium ions were stored in these organelles in MPS I lymphocytes. The differences between results of our research and those described in the cited study may result from using the splenic lymphocytes model by Pereira et al. (2010), while in this work, fibroblast lysates and all organ homogenates were investigated. Even so, these results are not necessarily mutually exclusive, but they might indicate that calcium accumulates in some organelles in MSP I cells, while Ca^2+^ levels in other parts of the cell and in extracellular matrix remain significantly lower.

The decreased levels of metal ions are not often observed in relation to human diseases. Studies on many of them showed rather increases in concentrations of metal ions correlating with some biochemical features of diseases, but it turns out that this is not the rule. For example, data showing both a decrease and an increase in the level of metal ions were observed in the case of Alzheimer’s disease depending on the selected model, tissue or experimental set-up (Wang et al. 2020). So far, observations of reduced levels of metal ions have been reported in the case of chronic kidney disease, in which the decrease in Zn^2+^ concentration was correlated with growth disturbance, taste impairment, anorexia and loss of appetite, dermatitis, delayed wound healing, and infection (Nakatani et al. 2021). Moreover, lowered Ca^2+^ levels corelate with skeletal and cardiovascular disorders, muscle aches, hypertension, periodontal disease, or diabetes mellitus (Peterlik et al. 2009). All these symptoms are also observed in some MPS patients as secondary or tertiary effects. It is likely that the changes in the ionic balance found in this work contribute to the mentioned symptoms.

Due to the existence of extensive feedback mechanisms regulating the concentration of individual ions and the expression of genes encoding ion-related proteins (Rosati and McKinnon 2004), it is not entirely clear which of the phenomena observed in this study is the cause and which is the consequence. In addition to the regulation of calcium concentration in the cell by calcium channels, a well-known phenomenon is the regulation of the expression of genes encoding calcium channels as a result of the influx or efflux of Ca^2+^ (Buonanno and Fields 1999; Spitzer et al. 2000; Barbado et al. 2009) mediated by transcription factors (West et al. 2002; Hogan et al. 2003). It is one of the best-known feedback mechanisms in the regulation of ion concentration in cells. Similarly, under conditions of Zn^2+^ deficiency, genes encoding the transporter responsible for its export from the cell are down-regulated, thus its constant level in the cell is maintained (Takeda et al. 2001; Chowanadisai et al. 2005). In addition, the expression of genes encoding ion-binding proteins, and not just ion-transporting channels, can be regulated by the levels of the ions themselves. An example is the *MTF-1* gene product which, by binding Zn^2+^, regulates the expression of other genes related to zinc homeostasis, but also its own gene (Dong et al. 2015).

In the light of the examples described above, a similar feedback mechanism can be found in the disorders of Ca^2+^ concentration and the level of expression of the *POSTN* gene encoding periostin, described in this report. Some studies indicated that periostin increases the level of calcium, and thus, plays a significant role in bone calcification (Zhu et al. 2021). Other works indicated, however, that the lack of periostin may lead to excessive calcium deposition in the bones (Tkatchenko et al. 2009). Obtaining such varied data may result from the selection of a different tissue for research, i.e. arteries or aortic valves. On the other hand, it has been reported that a reduced Ca^2+^ uptake leads to an increase in the level of the *POSTN* gene expression (Lombardi et al. 2019). Therefore, it is difficult to assess whether in the case of MPS a reduced level of calcium ions or an increased level of *POSTN* expression initiates a cascade of disorders. Nevertheless, it is certain that the resulting changes in skeletal calcification are one of the most bothersome symptoms of MPS, mainly in type IV (McClure et al. 1986; Marek et al. 2021). It cannot be ruled out that changes in the expression level of other genes coding for ion-binding proteins, detected in this study, are also caused by disturbances in the concentrations of metal ions. However, the exact regulatory mechanisms of interactions between individual metal ions and the expression of genes encoding ion-binding proteins in the pathogenesis of MPS have yet to be investigated.

One of the causes of disturbances in the levels of calcium ions may also be their decreased or increased efficiency of import/export from and to the cell. The genes whose products create ion channels and whose expression disturbances have been observed in this study include, between others, *TMEM38B*. Reduced expression of the *TMEM38B* gene, which encodes a protein that forms the intracellular calcium channel, may negatively affect the level of calcium in the cell, and thus, impair calcium-dependent signaling. Deletion mutations of this particular gene have also been reported in the rare disease osteogenesis imperfecta (OI) which is caused by defects in proteins involved in collagen interactions (Volodarsky et al. 2013; Cabral et al. 2016; Ramzan et al. 2021). It turns out that patients with OI are often clinically similar to patients with MPS. Cases have been reported in which, after a preliminary diagnosis of MPS, as a result of genetic testing, it turned out that patients suffered from OI associated with mutations in collagen-coding genes (*COL1A2*), while no mutations in the lysosomal genes were identified (Mutlu et al. 2015; Hamatani et al. 2018). This is even more interesting as disturbances in the expression of collagen-related genes (*COL8A2, COL5A1*) have also been identified in MPS (Gaffke et al. 2020). Therefore, it is probable that changes in both connective tissue and bone system in OI and MPS arise from disturbances in the functioning of the calcium channel (as the sole factor or one of the pathogenic factors). Although the reduction in the expression of the *TMEM38B* gene is not the result of a mutation, but rather one of the secondary effects of disturbances in cell metabolism, further studies on the functioning of this calcium channel are required. Such studies would help to decide whether MPS should be classified as a chanellopathy or not.

The results obtained in this study may suggest the need to find a way to stimulate the efflux of various ions from cellular organelles. Alternatively, one might suggest to include supplementation with Ca, Fe or Zn, or with compounds that would increase their concentration in the body. A combination therapy approach has already been proposed; such a therapy consists of ERT supplemented with compounds targeting cartilage and bone disorders. The experimental therapy in the rat MPS VI model, in which animals treated with ERT in combination with infliximab showed improved bone length and better motor performance compared to animals receiving ERT alone, looks promising. Similarly, in rats with MPS I and VI treated with ERT and pentosan polysulfate, an improvement in in the tracheal, skull and dentition disorders was observed as compared to rats treated with enzyme alone. Pentosan polysulfate was also administered to patients receiving ERT, resulting in a reduction in GAG levels and an improvement in joint mobility (Fecarotta et al. 2018). Thus, the creation of a combination therapy based on supplementation targeting ion disorders together with ERT could similarly have a positive effect on the course of MPS.

## Conclusions

Changes in the expression of genes responsible for maintaining the ionic balance and the coexisting disturbances in the concentrations of selected ions (Ca^2+^, Fe^2+^, Zn^2+^) in cells derived from MPS patients and in tissues of the MPS I mouse model indicate a significant role of disturbed ions’ homeostasis in the pathogenesis of MPS. Thus, one might suggest to establish the recovery on ionic balance as a new therapeutic target, supporting the currently used and not fully effective ERT.

## Supplementary Information

ESM 1 (PDF 906 kb)

## Data Availability

The RNA-seq raw results have been deposited in the NCBI Sequence Read Archive (SRA) (https://www.ncbi.nlm.nih.gov/sra), and they are available at the accession no. PRJNA562649. Other raw results are available at request from the authors.

## Abbreviations

ERT: Enzyme replacement therapy
FC: Fold change
GAG: Glycosaminoglycan
HDFa: Human Dermal Fibroblasts, adult
MPS: Mucopolysaccharidosis
