# Supplementary material for: Impaired ion homeostasis as a possible associate factor in mucopolysaccharidosis pathogenesis: transcriptomic, cellular and animal studies
Source: Metab Brain Dis. 2021 Dec 20;37(2):299–310. doi: 10.1007/s11011-021-00892-4 (PMC8784502; doi:10.1007/s11011-021-00892-4)
Supplement: Supplementary file 1 — (PDF 906 kb) [file 11011_2021_892_MOESM1_ESM.pdf]

Supplemental Material

Fig. S1

ion transport

No. of transcripts with individual log$_2$FC values in given MPS types/subtypes relative to control cells

	I	II	IIIA	IIIB	IIIC	IIID	IVA	IVB	VI	VII	IX
log$_2$FC>4,5	-	-	-	-	-	-	-	-	-	-	-
log$_2$FC>3,5	-	-	-	-	1	-	-	-	-	-	-
log$_2$FC>2,5	-	-	-	2	3	-	-	2	1	1	2
log$_2$FC>1.5	1	1	6	8	11	2	2	6	2	8	5
log$_2$FC<-1,5	3	1	2	7	5	6	-	10	2	5	7
log$_2$FC<-2,5	2	-	1	2	-	-	-	1	-	1	1
log$_2$FC<-3,5	1	-	1	1	-	-	-	1	-	-	-
log$_2$FC<-4,5	1	-	1	-	-	-	-	1	-	-	-

ion homeostasis

No. of transcripts with individual log$_2$FC values in given MPS types/subtypes relative to control cells

	I	II	IIIA	IIIB	IIIC	IIID	IVA	IVB	VI	VII	IX
log$_2$FC>4,5	-	-	-	1	1	-	-	1	-	-	-
log$_2$FC>3,5	-	-	1	1	2	1	-	1	-	-	2
log$_2$FC>2,5	-	-	1	5	3	1	1	3	-	3	3
log$_2$FC>1.5	3	1	5	7	6	4	2	7	-	8	6
log$_2$FC<-1,5	5	-	4	4	4	2	1	6	-	2	3
log$_2$FC<-2,5	2	-	2	1	-	-	-	1	-	1	1
log$_2$FC<-3,5	1	-	1	1	-	-	-	1	-	0	-
log$_2$FC<-4,5	1	-	1	-	-	-	-	1	-	0	-

Fig. S1. Number of transcripts included into ‘ion transport’ (GO:0006811) and ‘ion homeostasis’ (GO:0006873) terms with altered expression depending on the level of fold-change value (log$_2$FC) in different types/subtypes of MPS relative to control cells (HDFa).

Fig. S2

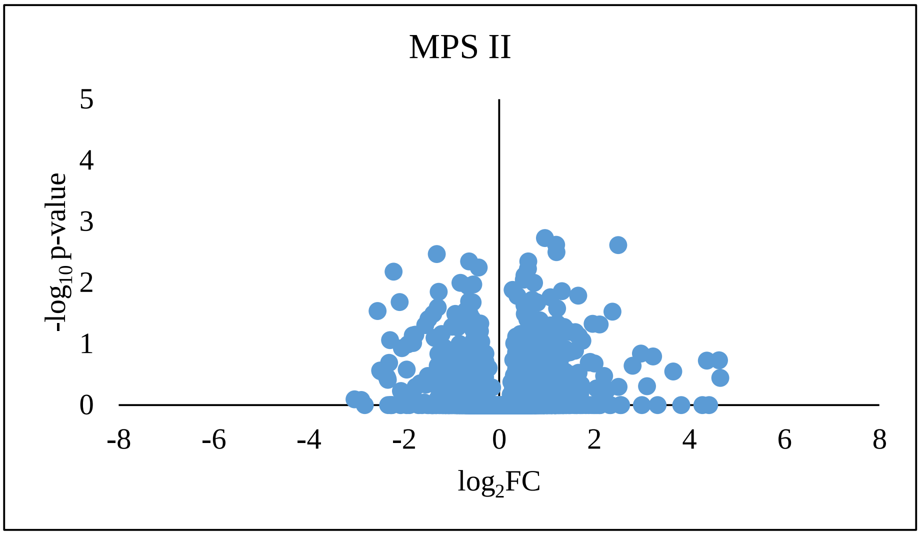

Fig. S2. Volcano plots indicating the lack of transcripts which significantly changed expression ($log_2FC > 3.5$) included into ‘ion binding’ (GO:0043167) term in the case of MPS II relative to control cells (HDFa).

Fig. S3

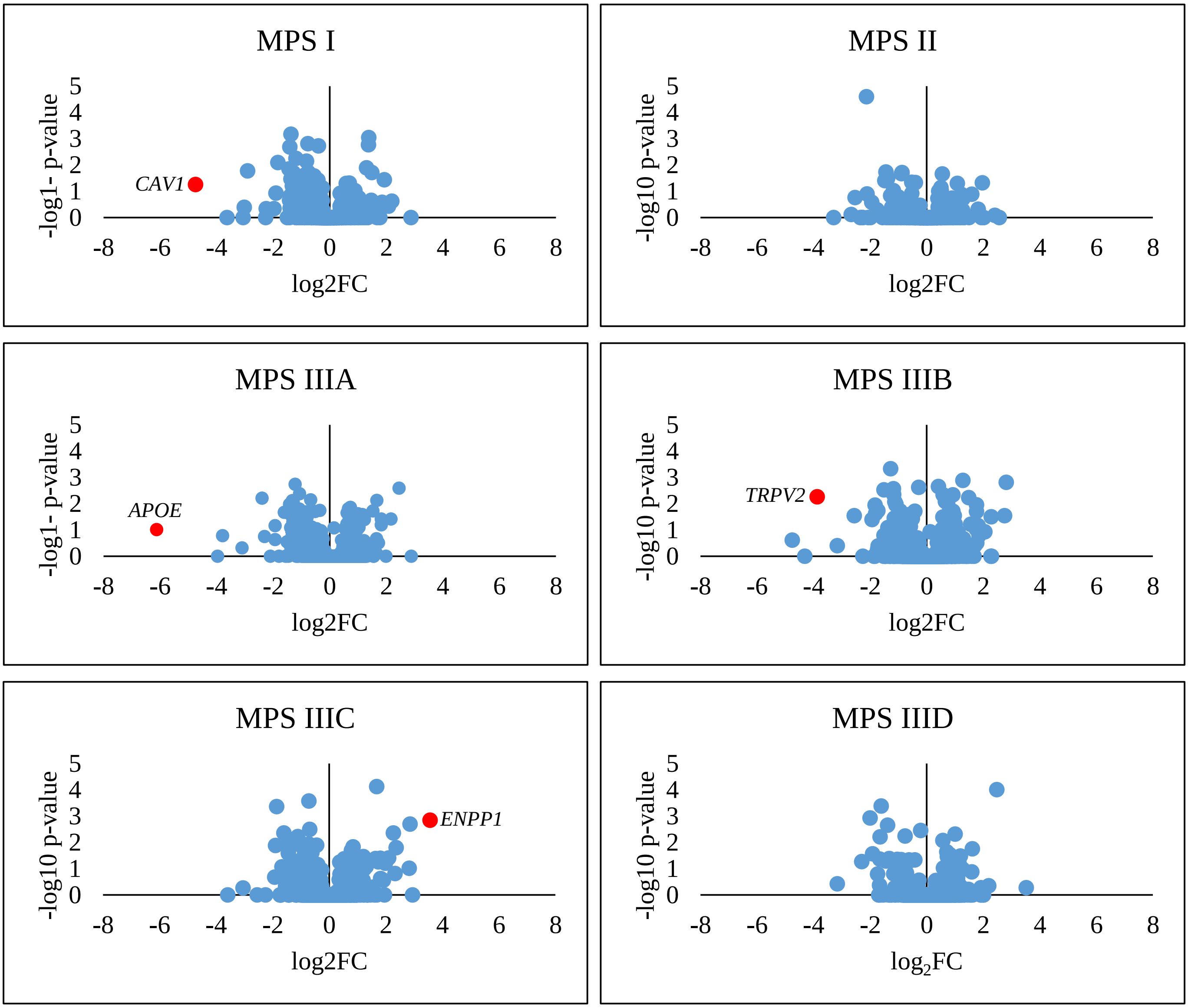

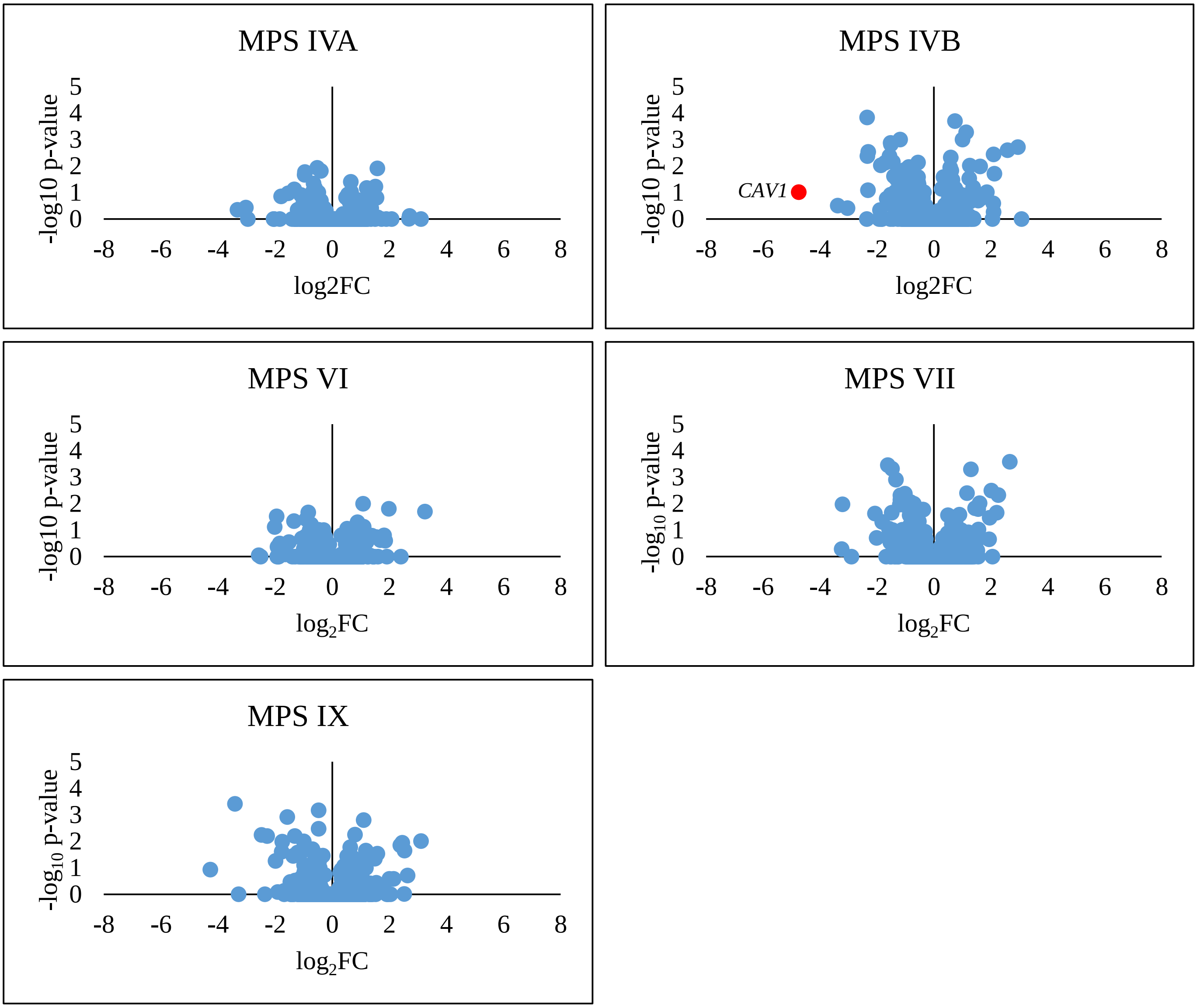

Fig. S3. Volcano plots indicating transcripts which expression is significantly changed ($log_2FC > 3.5$) included into ‘ion transport’ (GO:0006811) term in different types/subtypes of MPS relative to control cells (HDFa).

Fig. S4

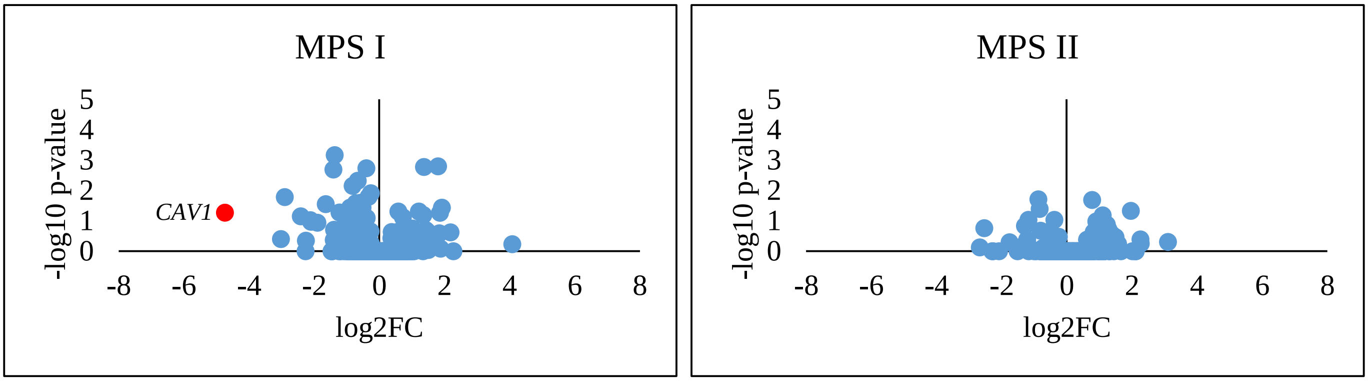

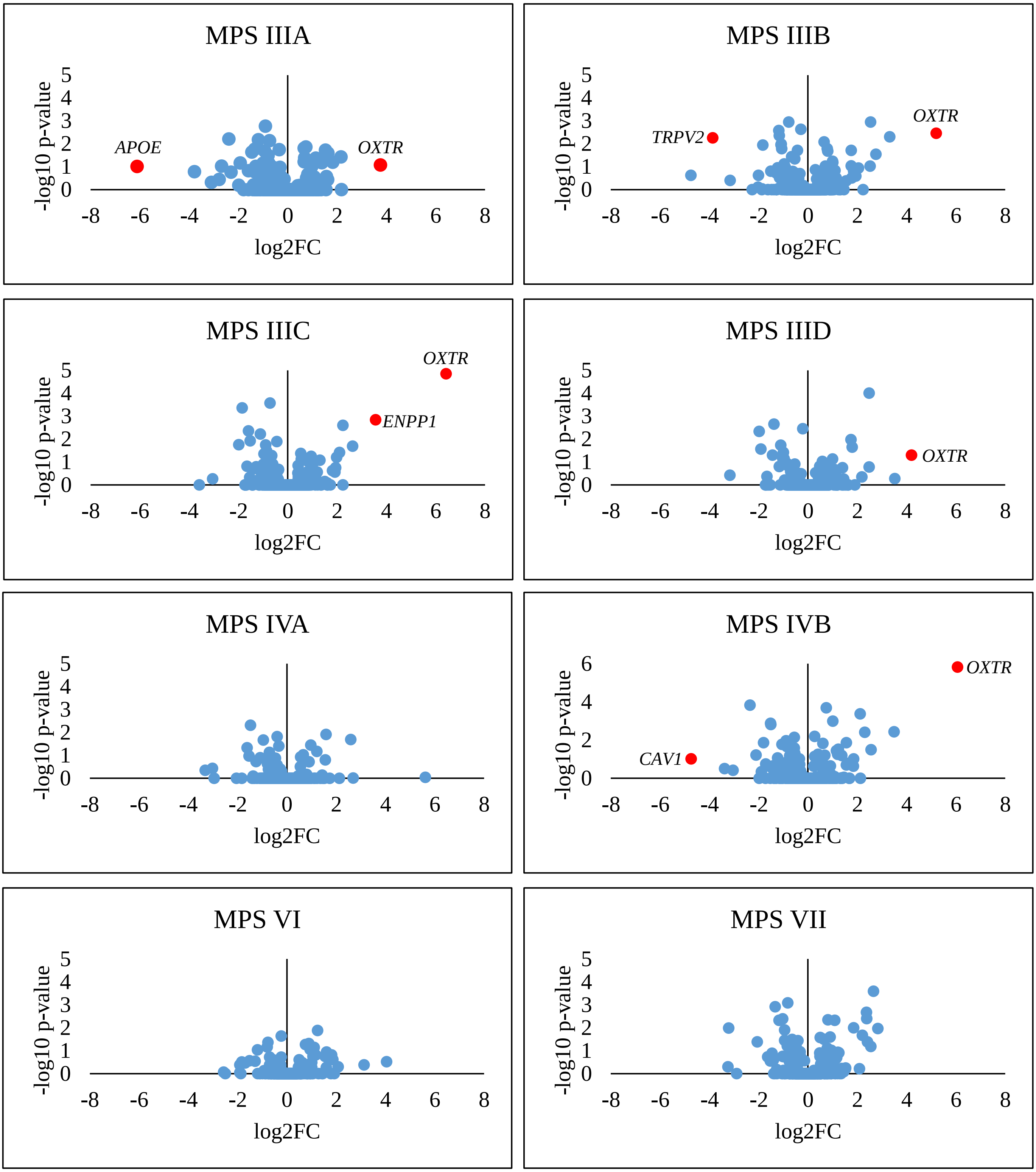

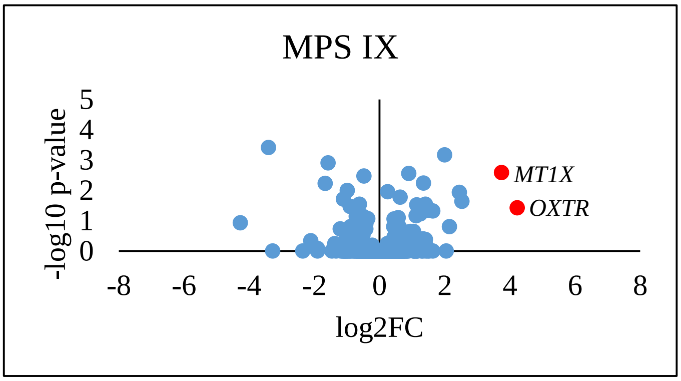

Fig. S4. Volcano plots indicating transcripts which expression is significantly changed ($\log_2$FC > 3.5) included into 'ion homeostasis' (GO:0006873) term in different types/subtypes of MPS relative to control cells (HDFa).
